# Adherence to the Mediterranean Diet during Pregnancy: Associations with Sociodemographic and Anthropometric Parameters, Perinatal Outcomes, and Breastfeeding Practices

**DOI:** 10.3390/medicina59091547

**Published:** 2023-08-25

**Authors:** Georgios Antasouras, Sousana K. Papadopoulou, Olga Alexatou, Dimitrios Papandreou, Maria Mentzelou, Athanasios Migdanis, Evmorfia Psara, Ioannis Migdanis, Maria Chrysafi, Stefanos Tyrovolas, Aikaterini Louka, Constantinos Giaginis

**Affiliations:** 1 Department of Food Science and Nutrition, School of the Environment, University of the Aegean, Myrina, 81400 Lemnos, Greece; g.antasouras@gmail.com (G.A.); rd.olga.alexatou@gmail.com (O.A.); maria.mentzelou@hotmail.com (M.M.); fnsd21013@fns.aegean.gr (E.P.); m.chrisafi3@gmail.com (M.C.); loukathy612@gmail.com (A.L.); cgiaginis@aegean.gr (C.G.); 2 Department of Nutritional Sciences and Dietetics, School of Health Sciences, International Hellenic University, 57400 Thessaloniki, Greece; 3 Department of Clinical Nutrition & Dietetics, College of Health, University of Sharjah, Sharjah P.O. Box 27272, United Arab Emirates; dpapandreou@sharjah.ac.ae; 4 Department of Gastroenterology, Faculty of Medicine, University of Thessaly, 41500 Larissa, Greece; thanmig@yahoo.com; 5 Department of Nutrition and Dietetics, University of Thessaly, 42132 Trikala, Greece; johnymig@hotmail.com; 6 School of Nursing, The Hong Kong Polytechnic University, Hung Hom, Kowloon GH506, Hong Kong; stefanos.tyrovolas@polyu.edu.hk

**Keywords:** Mediterranean diet, pregnancy, pre-pregnancy obesity, gestational diabetes, gestational hypertension, sociodemographic characteristics, anthropometric characteristics, perinatal outcomes, type of delivery, breastfeeding

## Abstract

*Background and Objectives*: The Mediterranean diet (MD) has been recognized as a beneficial nutritional pattern that promotes human health, decreasing the risks of a variety of human disorders and pathological states, including adverse pregnancy outcomes. In this aspect, the current survey aimed to assess the potential association of compliance with the MD during gestation with various sociodemographic and anthropometric parameters, perinatal outcomes, and breastfeeding practices. *Materials and Methods*: This was a cross-sectional study performed on 5688 pregnant women from 10 distinctive Greek areas. Face-to-face interviews with qualified questionnaires and thorough retrievals of medical records were performed to collect data concerning the participants’ sociodemographic and anthropometric parameters, perinatal outcomes, and breastfeeding practices. *Results*: Elevated compliance with the MD during pregnancy was independently related with older age, higher educational status, and better economic status as well as decreased incidences of pre-pregnancy overweight/obesity and excess gestational weight gain and a lower likelihood of gestational diabetes. Moreover, greater adherence to the MD was independently associated with an increased prevalence of delivering vaginally and a greater prevalence of exclusive breastfeeding for at least 16 weeks postpartum. *Conclusions*: A higher level of compliance with the MD for the period of gestation was associated with several favorable lifestyle factors that may promote maternal health. Further studies with a prospective design as well as studies exploring the potential effects of maternal compliance with the MD for the period of pregnancy on the health of children should be performed. Future studies should also be extended beyond the MD by assessing the potential beneficial effects of adopting a Mediterranean lifestyle on maternal and child health.

## 1. Introduction

The Mediterranean diet (MD) is a plant-based diet comprising high amounts of vegetables, fruit, cereals, nuts and extra virgin olive oil, a moderate consumption of dairy products, fish, and poultry, and a low intake of saturated fats and processed red meat products. The MD was primarily invented by Ancel Keys in 1960 [1]; it has been considered one of the most-investigated and broadly admired diets and has reasonably attracted the worldwide interest of the scientific community [2]. This is ascribed to the fact that the MD has been increasingly found to exert diverse beneficial effects on human health, promoting great daily quality of life and healthy aging and increasing life expectancy [3]. Several studies have provided substantial evidence that the MD can function as a preventative agent, acting against a variety of human disorders by lowering the risks of cardiovascular diseases, metabolic disorders, neurodegenerative diseases, cancer, and other human diseases [4,5,6,7]. Regarding anthropometric parameters, Mediterranean diet-based interventions can also prevent obesity and obesity-related diseases [8,9,10]. However, even the residents of the Mediterranean basin have been found to adopt the MD only moderately in the last decade, highlighting the need to increase the promotion of compliance with the MD, at least in the countries of its origin and additionally in the countries beyond them [11].

Diet and the state of nourishment have been considered crucial factors for the health of both pregnant women and their fetuses during gestation [12]. The MD can promote the maintenance of female reproductive health, exerting an important positive impact by preventing and monitoring reproductive health disorders in women [13]. Moreover, newborns delivered by women that greatly adopted the MD during gestation also show greater neurodevelopment and better anthropometric indexes [14]. Remarkably, a recent cohort study suggested that strong compliance with the MD may reduce the risk of harmful gestational complications in a dose–response manner [15]. Thus, the pattern of the MD seems to exert an overall crucial impact in promoting women’s health throughout their lifetimes, including the period of gestation.

A high level of compliance with the MD has been shown to decrease the risk of developing gestational diabetes. In parallel, it can promote glucose tolerance to a higher extent, even in pregnant women with an absence of gestational diabetes [16,17]. Better compliance with the MD has been associated with reduced odds of pregnancy-induced hypertension and pre-eclampsia [18]. Additional studies have documented that adopting healthier nutritional patterns, including the MD, during both the periconceptional and gestational periods may reduce the risk of developing gestational diabetes and/or hypertension, pre-eclampsia, and preterm birth [19]. Adherence to the MD was also related to a lower risk of excess gestational weight gain (GWG), while optimal adherence to the MD may slow down iron deficiency and/or anemia during gestation [20].

Furthermore, a recent systematic review demonstrated that adherence to the MD was associated with decreases in several pathological states developed during gestation, such as gestational diabetes, overweight/obesity, sleep disturbances, complications during delivery, urinary tract infections, and fetal development difficulties, as well as perinatal outcomes including childbirth weight, prematurity, and gastroschisis [21]. A high level of compliance with the MD seems to prevent the maternal gain of excess body weight during gestation, maintaining GWG at the recommended levels [22]. Strong compliance with the MD has also been identified as a critical lifestyle approach to preventing adverse pregnancy outcomes, especially in older pregnant women who exhibit higher levels of risk for adverse pregnancy outcomes [23].

Several studies have provided evidence that strong compliance with the MD during gestation can reduce several cardiometabolic risk factors like high blood pressure and adiposity [24]. A greater level of compliance with the MD has been associated with advanced age, a higher prevalence of normal BMI status, and better physical fitness, which promote physical activity recommendations [25]. A reduced intake of red meat and sweets and a simultaneously higher consumption of whole grain cereals, fruits, vegetables, seafoods, olive oil, and nuts, have been found to promote greater mental health during gestation, such as a decrease in negative affect, reductions in the prevalence of the symptoms of anxiety and depression, and improved emotional monitoring, resilience, and positive affect during the period of pregnancy [26]. A recent prospective study also showed that the MD may reduce GWG and postpartum excess weight retention, underlining the significance of encouraging the adoption of the MD, which may exert several health benefits for both a mother and her child [27].

The impact of maternal diet has been interrelated with several lifestyle factors such as smoking, BMI status, educational level, economic status, age, and place of residency [28]. Being older with an advanced educational level, maintaining a normal BMI, and avoiding smoking have been linked with higher levels of consumption of fruits, vegetables, seafoods, whole-grain foodstuffs, probiotics, and vegetarian dishes, having salad as a main meal, and a greater intake of vitamins and minerals [29,30,31]. In contrast, having an excess BMI and being a systematic smoker before pregnancy have been related with the intake of white bread, candy, snacks, fried foodstuffs, soft drinks, red meat and its processed products, milk, and pizza [29,30,31]. Moreover, women who comply greatly with the MD were more often characterized by a GWG that meets the recommendations of the International Organization of Medicine (IOM) [32,33].

Overall, the favorable impact of the MD on the health of a woman throughout the period of pregnancy, and the significance of a pregnant woman’s health for her infant later in its life, have attracted the interest of the scientific community; however, there is currently no strongly conclusive evidence concerning specific healthy dietary patterns, such as the MD [34]. Among postpartum women, 15–20% maintain ≥5 kg of their GWG, enhancing the probability of overweight or obesity in the next years of their life [34]. In this aspect, breastfeeding can reduce excess maternal weight gain during pregnancy and can also decrease the mother’s likelihood of developing breast and ovarian cancers, hypertension, and insulin-independent diabetes [35,36]. Another study also revealed that higher adherence to the MD across gestational and breastfeeding periods has been shown to reduce the gain of excess body weight which was obtained during pregnancy, decreasing the risk for mothers of being overweight or obese in the first years following delivery [37].

However, there are no adequate and reliable data thus far concerning the probable relation of compliance with the MD during gestation with diverse maternal sociodemographic and anthropometric parameters, perinatal outcomes, and breastfeeding practices. Most of the existing evidence has explored the impact of adhering to the MD in the pre-pregnancy period, while the existing evidence on adherence to the MD during gestation still remains scarce worldwide. In this aspect, this cross-sectional study intents to investigate whether adherence to the MD during pregnancy could be related to sociodemographic and anthropometric parameters, perinatal outcomes, and breastfeeding practices in a representative population of reproductive-age women in Greece.

## 2. Materials and Methods

### 2.1. Study Population

In the current survey, 8086 pregnant Caucasian women in the third trimester of gestation were assigned primarily from 10 different regions in Greece, including Athens, Thessaloniki, Larissa, Patra, Alexandroupolis, Kalamata, Larissa, Ioannina, Crete, and the North and South Aegean, including both urban and rural regions. The assignment of patients to the study was performed from May 2016 to September 2020. Among the initially assigned 8086 Caucasian women, 128 of them primarily agreed to take part to the survey but later decided not to participate to the study. In multiparous participating women, only the last pregnancy was included in this study.

All the collected data were strictly confidential. The assigned women were informed concerning the purposes of the study, and they each endorsed an approval form on which they agreed that their individual data would be published anonymously. The calculation of the sample size was performed utilizing PS, the Power and Sample Size calculator program, in order to obtain sufficient power for to examine the hypothesis [38,39].

**Figure 1:**
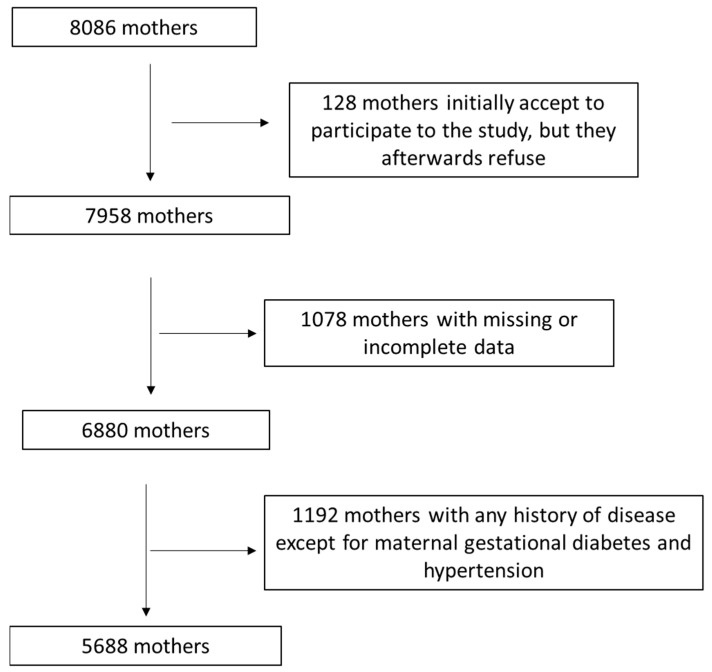
Flow chart of study enrolment.

Amongst the remaining 7958 assigned women, 1078 of them (13.7%) were not included in the current analysis due to lost or incomplete data. Amongst the rest of the 6880 women, 1192 (17.3%) were excluded from the current analysis due to a history of disease like diabetes mellitus types 1 or 2, dyslipidemia, increased arterial tension, anemia, thyroid diseases, cardiovascular disorders, osteoporosis, multiple sclerosis, polycystic ovary syndrome, inflammatory gastrointestinal disorders, gallstones, autoimmune liver disorder, celiac disorder, pelvic floor disfunction, and tumor malignancies. Finally, 5688 women were enrolled in the final analysis by applying the above inclusion/exclusion criteria, resulting in a final response rate of 70.3%. In the enrolled women, the presence of a previous disorder was assessed via self-reporting. Gestational diabetes and gestational hypertension were the only diseases that were included in the present study. A flow chart of enrolment for the study is presented in Figure 1.

The present study was approved by the Ethics Organization Committee of the University of the Aegean (ethics approval protocol no. 12/14.5.2016) and was in accordance with the World Health Organization (52nd WMA General Assembly, Edinburgh, Scotland, 2000).

### 2.2. Study Design

Semi-quantitative questionnaires were applied to evaluate the sociodemographic characteristics and perinatal outcomes of the enrolled pregnant women. Each participant’s weight in the initial weeks of gestation and immediately prior to the childbirth were recovered from their personal medical records. Body weight and height were measured during the patients’ first visits to health care units [38,39]. Each mother’s pre-pregnancy BMI status was classified based on the measured weight and height derived from medical records. Each mother’s weight was assessed using the same electronic scale, and height was evaluated using a portable stadiometer. The WHO method was utilized to categorize overweight and obesity in the enrolled women [40]. GWG was estimated by subtracting the recovered determined weight from the initial weeks of gestation by the recovered determined body weight immediately prior to childbirth. Based on Institute of Medicine (IOM)’s guidelines, the recommended gestational weight gain for women who were underweight prior to pregnancy (BMI < 18.5 kg/m^2^) ranges from 12.5 to 18.0 Kg; for normal weight women (BMI: 18.5–24.9 kg/m^2^), the GWG ranges from 11.6 to 16.0 Kg; and for overweight women (BMI: 25.0–29.9 kg/m^2^) and obese women (BMI ≥ 30.0 kg/m^2^), the GWG ranges from 7.0–11.5 Kg [41,42].

The mothers’ gestational diabetes diagnoses were recovered from their medical records. All the mothers were tested for gestational diabetes mellitus using a standardized oral glucose tolerance test (OGTT) during gestation [43]. Specifically, a fasting OGTT with 75 g of glucose with a cut-off plasma glucose level of ≥140 mg/dL after 2 h for the first trimester and the following trimester at 24–28 weeks of pregnancy were performed for all participating mothers [43]. Accordingly, the data concerning pregnancy-induced hypertension were recovered from the patients’ medical records.

Moreover, the assigned pregnant women provided information as to whether they adopted breastfeeding practices at all and if they exclusively breastfed their infant for at least 4 months. To minimize recall bias, the women responded whether they adopted an exclusive breastfeeding practice for at least 4 months as at this exact time, they were advised to gradually include pulp foodstuffs into the eating practices of their paired infants and they therefore remembered precisely this time point more precisely, rendering their answers more reliable. In contrast, women who attempted lactation for shorter periods were not able to respond with adequate reliability concerning the exact period of their breastfeeding practice.

The pregnant women were further asked whether they had a preterm birth (<37th week), and their answers were further cross-checked with their medical records to ensure more reliable data concerning the exact week of pregnancy during which preterm birth was performed. However, some records omitted the exact week of preterm childbirth, and some of them were not in accordance with the women’s answers; therefore, preterm birth was categorized as a binary variable, with childbirth categorized prior to or next to the 37th week of pregnancy.

The women’s age, educational and economic status, nationality, place of residence, parity, smoking habits, and type of delivery were retrieved via relevant self-reported questionnaires according to memory recall. Particularly, the educational level was categorized into three classes: (a) primary education, (b) secondary education, and (c) university studies. Economic status was classified based on the annual family income as EUR 0 < 5000; (1) EUR 5000–10,000; (2) EUR 10,000–15,000; (3) EUR 15,000–20,000; (4) EUR 20,000–25,000; and (5) >EUR 25,000. Economic status was further classified as low for a family yearly income of ≤ EUR 10,000, medium for a yearly income ˃EUR 10,000 and ≤EUR 20,000, and high for a yearly income of ˃EUR 20,000.

As far as adherence to the MD assessment is concerned, the validated MedDietScore was used [44,45]. This questionnaire evaluates the frequency of the consumption of food belonging to eleven selected groups of foodstuffs based on MedDietScore index. In each question there were six possible responses marked between 0 and 5, which depend on the level of adherence for each foodstuff group. The sum of the eleven responses results in a score between 0 and 55; a greater score represents a higher level of adherence to the MD [44,45]. Concerning cereals, potatoes, fruits, vegetables, dairy products, and olive oil, the rates of 6 probable responses are related to daily consumption. Regarding legumes, fish, red meat, and poultry, the rates of 6 probable responses were related to weekly consumption [44,45]. The 11th question evaluates wine drinking on a daily frequency, with intermediate drinking (≤1 and ≤2 drinks/day for women and men, respectively; one drink = 100 mL = 12 g of ethanol) being recognized as the greatest score [44,45]. However, the last question was omitted from the analysis, as alcohol intake is not recommended during pregnancy.

Detailed clarifying instructions were systematically supplied to the enrolled women by trained personnel regarding the completion of the questionnaires, while a comprehensive presentation of the included questions was systematically provided to enhance the reliability of the women’s responses. Direct face-to-face interviews between participating women and trained personnel were performed concerning data such as the sociodemographic characteristics, which were not retrieved from the women’s medical files to minimize recall bias.

### 2.3. Statistical Analysis

Statistical analyses were performed using Student’s *t*-test for continuous variables, which were found to be normally distributed. The normality of the variables’ distribution was assessed via the Kolmogorov–Smirnov test. By applying the above test, all continuous variables were found to be normally distributed. The categorical variables were analyzed by the chi-square test. The mean value ± standard deviation (SD) was applied concerning the quantitative variables that were found to follow a normal distribution. Absolute or relative frequencies were applied for qualitative variables. A multivariate binary logistic regression analysis was applied to evaluate whether compliance with the MD may exert an independent impact on sociodemographic and anthropometric parameters, perinatal outcomes, and breastfeeding practices after an adjustment for multiple confounders such as maternal age, educational and economic status, nationality, nationality, type of residence, smoking habits, parity, pre-pregnancy BMI status, GWG, preterm birth, gestational diabetes, gestational hypertension, type of delivery, and exclusive breastfeeding. Differences were categorized as significant at *p* < 0.05 and a confidence interval of 95%. The statistical analysis of the survey data was performed via Statistica 10.0 software, Europe (Informer Technologies, Inc., Hamburg, Germany).

## 3. Results

### 3.1. Descriptive Statistics of the Study Population

The present survey enrolled 5688 mothers who were enrolled during the third trimester of gestation. Table 1 presents the descriptive statistics of the enrolled women. The mean age of the pregnant women was 35.1 ± 4.8 years (range: 19–46 years). Concerning maternal education levels, 23.2% of the mothers had received a primary education, 44.5% of them had completed a secondary education, and 33.3% had graduated from a university. Regarding the economic level, 45.6% of mothers reported a poor family yearly income, 45.7% exhibited a moderate family yearly income, and 8.7% showed an elevated yearly income. Regarding nationality, 96.0% were Greek and the remaining 4.0% were of other nationalities. Regarding place of residence, 67.3% of the enrolled women lived in urban regions, and the remaining 32.7% lived in rural regions. Of the participants, 25.6% smoked systematically prior to gestation, and of the rest, 74.4% did not smoke at all. In total, 59.2% of the participants had no children since this was their first pregnancy, and 40.8% of the mothers had experienced more than one pregnancy that had resulted in a child aside from the present pregnancy.

The BMI of the enrolled women before pregnancy was 22.9 ± 3.7 Kg/m^2^ (range: 17.9–37.8 Kg/m^2^). In fact, 18.6% of the women were classified as overweight, and 5.7% were classified as obese before the initiation of pregnancy. In total, a prevalence of 22.3% women experiencing overweight/obesity before pregnancy was found based on the participants’ BMI classifications. The mean GWG of the pregnant women was 13.9 ± 6.2 (range: 0–45 Kg). Preterm birth (<37th week) was recorded for 30.0% of the enrolled women. Additionally, 6.5% of the pregnant women developed gestational diabetes, and 4.1% of the enrolled women presented with pregnancy-induced hypertension.

More than half of the participating women (56.4%) gave birth via a caesarean section, and the other resting 44.6% delivered vaginally. Almost half of the enrolled pregnant women (49.5%) breastfed their newborns exclusively for at least four months (mean duration: 4.6 ± 1.7months), and 50.5% of them did not exclusively breastfeed for at least four months or did not breastfeed at all.

### 3.2. Adherence to the MD in Association with the Sociodemographic and Anthropometric Characteristics of the Enrolled Pregnant Women

Pregnant women of an advanced age showed significantly greater compliance with the MD than those of lower ages (Table 2, *p* < 0.0001). Indicatively, pregnant women presenting elevated compliance with the MD exhibited a mean age of 36.1 ± 4.7 years old, whereas women presenting less compliance with the MD were approximately two years younger, with a mean age of 34.4 ± 4.5 years (Table 2). Women with a higher level of education demonstrated substantially greater compliance with the MD than those with a reduced education status (Table 2, *p* ˂ 0.0001).

Pregnant women with higher levels of adherence to the MD had significantly better family economic statuses than those with decreased compliance with the MD (Table 2, *p* = 0.0087). Non-smoking pregnant women exhibited considerably greater compliance with the MD than women who smoked (Table 2, *p* = 0.0261). Pregnant women living in rural regions had marginally higher levels of compliance with the MD than those living in urban regions, although at a non-significant level (Table 2, *p* = 0.0576). The nationality and parity of the women did not show any considerable relation or correlation with compliance with the MD (Table 2, *p* = 0.1015 and *p* = 0.2125, respectively).

Pregnant women with higher levels of compliance with the MD had normal-weight BMI values significantly more frequently prior to pregnancy than those with lower levels of compliance with the MD (Table 2, *p* < 0.0001). Indicatively, among the pregnant women with high levels of adherence to the MD, 14.7% of them were classified as overweight or obese. This prevalence was doubled among the pregnant women with very low adherence to the MD, as 28.3% of them were affected by overweight or obesity (Table 3).

### 3.3. Adherence to the MD in Relation to the Perinatal Outcomes of the Enrolled Women

The pregnant women with greater adherence to the MD had significantly lower GWG rates compared to those with lower compliance with the MD (Table 2, *p* < 0.0001). Indicatively, women presenting with elevated adherence to the MD exhibited a mean GWG value of 13.4 ± 5.9 Kg, whereas those with decreased compliance with the MD had an elevated GWG value of 14.5 ± 6.5 Kg, i.e., about more than 1 Kg (Table 2). The pregnant women with elevated compliance with the MD exhibited a considerably lower risk of developing gestational diabetes than the women with lower levels of adherence to the MD (Table 2, *p* < 0.0001). Characteristically, among the women with very low levels of adherence to the MD, 10.8% of them were diagnosed with gestational diabetes, whereas this prevalence was considerably decreased among the women with high levels of adherence to the MD, as only 3.2% of them developed gestational diabetes (Table 2). Preterm birth and pregnancy-induced hypertension did not show any significant association or tendency with compliance with the MD (Table 2, *p* = 0.1943 and *p* = 0.2011, respectively).

### 3.4. Relation of Adherence to the MD with Type of Delivery and Breastfeeding of the Study Population

Pregnant women presenting with elevated adherence to the MD delivered vaginally significantly more frequently compared to those with lower compliance with the MD (Table 2, *p* < 0.0001). Indicatively, amongst the women presenting elevated compliance with the MD, 41.5% of them delivered via caesarean section, whereas this prevalence was considerably increased among the women presenting with very low levels of adherence to the MD, as 66.5% of them delivered via caesarean section (Table 2).

Women with higher levels of adherence to the MD exhibited s exclusive breastfeeding for a minimum period of four months significantly more frequently than those presenting with lower levels of adherence to the MD (Table 2, *p* < 0.0001). Characteristically, among the women with high levels of adherence to the MD, 82.2% of them adopted an exclusive breastfeeding practice for their newborns for a minimum period of four months, whereas this prevalence was considerably decreased among the women with very low adherence to the MD, as only 15.4% of them adopted an exclusive breastfeeding practice for their newborns for a minimum period of four months (Table 2).

### 3.5. Multivariate Regression Analysis for Adherence to the MD with Adjustment for Multiple Confounding Factors Such as Sociodemographic, Anthropometric, and Lifestyle Factors and Perinatal Outcomes

In the multivariate binary logistic regression analysis, levels of adherence to the MD were related at independent levels with the age, educational level, economic status, pre-pregnancy BMI and GWG, gestational diabetes, type of delivery, and breastfeeding practices of the women (Table 3). Pregnant women presenting elevated adherence to the MD exhibited a 28% greater probability of being older than those with lower adherence to the MD (Table 3, *p* = 0.0021). Pregnant women with higher adherence to the MD had an 88% greater likelihood of presenting with a higher educational level than those presenting lower levels of adherence to the MD (Table 3, *p* = 0.0032). Pregnant women showing greater adherence to the MD were associated with 36% greater odds of having a better economic status than those with lower adherence to the MD (Table 3, *p* = 0.0287).

Pregnant women with higher levels of adherence to the MD had more than a twofold lower probability of being overweight or obese before gestation than women with lower levels of adherence to the MD (Table 3, *p* = 0.0001). Pregnant women presenting greater compliance with the MD exhibited a 78% decrease in the probability of excess GWG than women presenting with decreased adherence to the MD (Table 3, *p* = 0.0007). Pregnant women presenting decreased adherence to the MD had a more than twofold higher probability of developing gestational diabetes than women presenting with lower levels of compliance with the MD (Table 3, *p* = 0.0004).

Pregnant women presenting greater compliance with the MD exhibited an 89% greater likelihood of giving a birth via vaginal delivery than women presenting reduced adherence to the MD (Table 3, *p* = 0.0003). Women with higher adherence to the MD exhibited a more than twofold greater probability of breastfeeding their newborns exclusively for at least four months than those with lower levels of compliance with the MD (Table 3, *p* = 0.0001).

## 4. Discussion

In the present survey, elevated levels of maternal compliance with the MD during pregnancy were related at independent levels with an advanced age, higher education level, and better economic status. Elevated level of maternal compliance with the MD throughout gestation was also related at an independent level with a decreased probability of receiving a gestational diabetes diagnosis. Moreover, elevated compliance with the MD was associated at independent levels with a higher prevalence of giving a birth via vaginal delivery and a greater incidence of adopting exclusive breastfeeding for a minimum period of four months postpartum. Smoking habits and type of residence were also identified as markers of compliance with the MD in a univariate analysis. Nevertheless, their effects on compliance with the MD were substantially attenuated by adjusting for potential confounders and were not significant in a multivariate analysis.

### 4.1. Adherence to the MD and Gestational Diabetes

In accordance with the present study, a recent longitudinal study performed on 743 pregnant women in Greece indicated that a greater compliance with the MD prior to gestation, specifically with a lower meat intake, could exert a preventative impact on the incidence of developing gestational diabetes independently of body weight status and other well-known risk factors prior to gestation [46]. A previous observational study performed on 1076 consecutive pregnant women from ten Mediterranean countries, including Greece, also found that a higher level of adherence to the MD was related to a decreased prevalence of gestational diabetes and a greater degree of glucose tolerance [16]. Another recent cross-sectional observational study of 193 low-risk women admitted to a private maternity hospital in Greece for delivery documented that a greater compliance with the MD throughout gestation was related with a lower risk of gestational diabetes [17].

### 4.2. Adherence to the MD and Pre-Pregnancy Overweight/Obesity, Gestational Hypertension, and Pre-Eclampsia

A well-designed longitudinal study conducted on 82 women who gave preterm singleton birth at post conceptional age (≤34 weeks) indicated that mothers with low adherence to the MD exhibited a significantly higher pre-pregnancy BMI and an elevated prevalence of overweight/obesity and gestational hypertension/pre-eclampsia [47]. Accordingly, this study showed that decreased compliance with the MD was related to pre-pregnancy maternal overweight and obesity but not with pregnancy-induced hypertension and/or pre-eclampsia [47]. In accordance with our results, a meta-analysis on seven studies showed that Mediterranean-style diet interventions did not decrease the prevalence of pregnancy-induced hypertension and/or pre-eclampsia in healthy pregnancies [48]. In contrast, a longitudinal multicenter survey on 7798 racially, ethnically, and geographically differential women with singleton gestations showed that women with high levels of adherence to the MD had a 28% reduced probability of pre-eclampsia or eclampsia and a 37% decreased risk of gestational diabetes [15]. These discrepancies regarding pre-eclampsia or eclampsia may be ascribed to the fact that adherence to the MD in the previous study concerning the period before gestation included women with singleton gestations and different races, ethnicities, and geographical residences [15]. In a prospective and longitudinal study on 1887 pregnant women, adherence to the MD throughout the periconception and gestational period was associated with reduced probabilities of gestational diabetes and pregnancy-induced hypertension, pre-eclampsia, and preterm birth [19]. The above survey agrees with our study concerning gestational diabetes but not with the other adverse pregnancy outcomes, which may be ascribed to the lower sample sizes of the previous studies.

### 4.3. Adherence to the MD and Sociodemographic and Lifestyle Factors

In accordance with our results, an older maternal age, advanced education level, and greater social status were related with elevated compliance with the MD pre-pregnancy, whereas an unhealthy lifestyle like smoking and a low frequency of exercise were related with reduced compliance with the MD [49]. A cross-sectional study conducted on 681 pregnant women also indicated that higher compliance with the MD was related with an advanced education level and better annual income [50]. Another study also reported that greater compliance with the MD was related to a better socioeconomical level and more frequent physical activity, while greater compliance with the MD was further related with living in an urban region [51]. In a more recent study, women who were older residents of a coastal region and who exhibited an advanced education degree and better annual income, as well as those with a decreased BMI, were associated with greater adherence to the MD [51]. Residents of rural regions were found to have higher levels of adherence to the MD compared to residents of urban regions in Cyprus [52,53]. In another study, it was shown that children living in rural areas exhibited higher levels of consumption of more traditional foods and ate fast food less frequently, being more likely to participate in meals with their families [54]. Guzeldere et al. documented that there was no considerable divergence concerning the adherence to the MD of women enrolled in their study based on their type of residency [55]. Accordingly, Torres et al. also reported that adherence to the MD was not associated with place of residence [56]. In the present study, we recorded only a marginal relation of compliance with the MD with the place of residence that did not remain significant after adjusting for multiple confounding factors. These conflicting results may be ascribed to the fact that the number of enrolled mothers living in rural areas is considerably lower than the number of mothers living in urban areas. In this aspect, further research is recommended to obtain conclusive evidence in this issue by including equal numbers of mothers living in urban and rural regions.

### 4.4. Adherence to the MD and Preterm Birth

Concerning preterm birth, we found no relation with Compliance with the MD during pregnancy. Across published studies, the results on the relationships of Adherence to the MD during pregnancy and birth outcomes such as preterm birth remained inconsistent. Some non-US prospective studies have reported opposing results: the risk of preterm birth was inversely related with higher pregnant women compliance to MD in one study but not in the other [57,58]. In fact, Mikkelsen et al., have documented that a higher Adherence to the MD throughout gestation could decrease the probability of prematurely childbirth in Danish women, whereas Meltzer et al., did not record any relation of Compliance with the MD with the probability of preterm birth in Norwegian women [57,58]. These discrepancies in the findings of the relation of Compliance with the MD with childbirth outcomes like preterm childbirth may be due to several factors, including participants characteristics, covariates controlled for each respective study statistical modeling, and divergencies in dietary assessment techniques. In addition, differences between the above studies may be ascribed to the different variables used in adjusted models, which may affect the final outcomes.

### 4.5. Adherence to the MD and Type of Delivery and Breastfeeding Practices

In addition, we found high rates of caesarean deliveries, as more than half of the participating women (56.4%) gave birth via caesarean section, while according to data from the WHO, a proportion of about 10% of all deliveries are usually performed via caesarean section, which is ideally only applied when there are strong medical recommendations [59]. This proportion dropped to 41.5% among the women with high levels of adherence to the MD. This finding may be ascribed to the fact that women with high levels of adherence to the MD usually attempt to have a healthier lifestyle by avoiding treatment with medication and surgical operations such as caesarean sections when it is feasible. Accordingly, we recorded higher rates of exclusive breastfeeding for a minimum period of 4 months in women with greater compliance with the MD during pregnancy, which also may be attributed to the adoption of a healthier lifestyle in which diet has a significant role. Moreover, women with high levels of adherence to the MD are more likely to adopt a healthy lifestyle such as the Mediterranean lifestyle [60], which may enhance their probability of preferring a normal delivery such as vaginal delivery and also selecting exclusive breastfeeding, which is advantageous for the health of the mother and child [61]. The Mediterranean lifestyle has the advantage of extending its daily habits outside an elementary nutritional pattern according to the interrelationships of the diverse characteristics of a healthy lifestyle [62].

### 4.6. Strengths of the Study

The current survey has several advantages as it was conducted on an adequate representative sample of pregnant women enrolled from diverse regions of Greece, including both urban and rural areas. The study population was quite large and included only Caucasian women living in ten different Greek regions, and its representativeness could be recognized as quite adequate. Hence, our findings could be generalizable in Caucasian European populations of other nationalities. Moreover, our study is one of the few studies that investigated the relation of compliance with the MD throughout gestation with several sociodemographic and anthropometrical characteristics, perinatal outcomes, and breastfeeding practices. Also, a simple randomization methodology was utilized which ensured the overall randomness of the enrolment of the participating woman to a specific group. In population-based clinical research like the current survey, this methodology can ensure the division of similar numbers of participants amongst groups. Another advantage of our study is that face-to-face interviews between the participating women and the qualified personnel were performed to reduce recall bias. The detailed explanation guidelines and the thorough demonstration of the questions that were provided during the face-to-face interviews may also minimize recall bias. Moreover, our study population contained only healthy pregnant women with no history of any severe disease. We also examined whether adherence to the MD during pregnancy exerted independent effects by adjusting for several potential confounding factors.

### 4.7. Limitations of the Study

The understanding of the current results should be taken into consideration with some limitations in mind. The cross-sectional design of the study reduces the likelihood of etiological conclusions, and the study suffers from a potential risk of recall biases, especially for the self-reported questions even if face-to-face interviews were performed. Thus, no definitive conclusions about causality should be derived due to our study design. However, the self-reported data were extensively applied in epidemiological studies, showing great consistency and validity for predicting several outcomes. Another disadvantage of our study concerns the fact that BMI was utilized to distinguish maternal overweight and obesity before gestation. Nevertheless, direct determinations of body fat mass and distribution are recommended to extend and validate our results. In addition, despite a thorough approach to confounding adjustment, we acknowledge the possibility for unmeasured confounding factors such as the mental health status and the physical activity of the participating women. Hence, although we have performed adjustments for several confounders, it is still probable that residual confounding may influence our results. Moreover, another limitation of our study is the fact that the enrolled mothers had an advanced age (mean age: 35.1 ± 4.8 years old). In this context, there is substantial evidence that pregnancy at an advanced maternal age (age > 35 years old) is considered a risk factor for adverse maternal and perinatal outcomes [63,64]. In the last few decades, pregnancies of advanced maternal age have become more prevalent, which may be associated with increased risks for adverse pregnancy outcomes for both the mother and the child [63,64].

## 5. Conclusions

The current survey is one of the few currently available studies that has investigated the relationship of compliance with the MD throughout gestation with sociodemographic and anthropometric characteristics, perinatal outcomes, and breastfeeding practices. Higher maternal levels of adherence to the MD during pregnancy were related in an independent manner with an older age, greater education level, and better economic status, a lower prevalence of pre-pregnancy maternal overweight/obesity, and lower risks of excess GWG and gestational diabetes, as well as a higher prevalence of vaginal delivery and the adoption of exclusive breastfeeding. In this aspect, further well-designed prospective studies should be performed to confirm the results of the current survey. Future studies exploring the potential relationships of maternal compliance with the MD throughout gestation with the health of children should also be performed. Future studies should be further extended beyond the MD by assessing the potential beneficial effects of adopting a Mediterranean lifestyle on the health of both the mother and child. Based on the present findings, the MD is highly recommended for the promotion of the health of both mothers and children. Future strategies and policies should inform not only the specific population of this study but also the general population for the beneficial effects of the MD in promoting public health.

## Figures and Tables

**Table 1:** Descriptive statistics of the study population.

Characteristics (*n* = 5688)	Descriptive Statistics
**Maternal age (mean ± SD; years)**	35.1 ± 4.8
**Maternal education level (*n*, %)**	
Primary education	1320 (23.2%)
Secondary education	2529 (44.5%)
University studies	1839 (32.3%)
**Family economic status (*n*, %)**	
Low	2592 (45.6%)
Medium	2601(45.7%)
High	495 (8.7%)
**Nationality (*n*, %)**	
Greek	5463 (96.0%)
Other	225 (4.0%)
**Place of residence (*n*, %)**	
Urban	3825 (67.3%)
Rural	1863 (32.7%)
**Smoking habits (*n*, %)**	
Non-smokers	4230 (74.4%)
Smokers	1458 (25.6%)
**Parity (*n*, %)**	
Nulliparity	3367 (59.2%)
Multiparity	2321 (40.8%)
**Pre-pregnancy BMI status (*n*, %)**	
Normal weight	4306 (75.7%)
Overweight	1060 (18.6%)
Obese	322 (5.7%)
**Gestational weight gain (mean ± SD; Kg)**	13.9 ± 6.2
**Preterm birth (<37th week; *n*, %)**	
No	3984 (70.0%)
Yes	1704 (30.0%)
**Gestational** **diabetes** **(*n*, %)**	
No	5316 (93.5%)
Yes	372 (6.5%)
**Pregnancy-induced hypertension (*n*, %)**	
No	5129 (95.9%)
Yes	219 (4.1%)
**Type of delivery (*n*, %)**	
Vaginal	2500 (44.6%)
Caesarean section	3188 (56.4%)
**Exclusive breastfeeding (*n*, %)**	
No	2874 (50.5%)
Yes	2814 (49.5%)

**Table 2:** Associations of Adherence to the MD with sociodemographic and anthropometric characteristics, perinatal outcomes, type of delivery, and breastfeeding practices.

Characteristics (*n* = 5688)	Adherence to the Mediterranean Diet				
	Very Low 1423 (25.02%)	Low 1420 (24.96%)	Moderate 1422 (25.00%)	High 1423 (25.02%)	*p*-Value
**Age (mean ± SD; years)**	34.4 ± 4.5	34.9 ± 4.6	35.6 ± 4.4	36.1 ± 4.7	*p* < 0.0001
**Education level (*n*, %)**					*p* < 0.0001
Primary education	746 (52.4%)	383 (27.0%)	104 (7.3%)	87 (6.1%)	
Secondary education	517 (36.3%)	852 (60.0%)	630 (44.30%)	530 (37.3%)	
University studies	160 (11.3%)	185 (13.0%)	688 (48.4%)	806 (56.6%)	
**Family economic status (*n*, %)**					*p* = 0.0087
Low	686 (48.2%)	598 (42.1%)	633 (44.5%)	675 (47.4%)	
Medium	640 (45.0%)	720 (50.7%)	623 (43.8%)	618 (43.4%)	
High	97 (6.8%)	102 (7.2%)	166 (11.7%)	130 (9.3%)	
**Nationality (*n*, %)**					*p* = 0.1015
Greek	1362 (95.7%)	1373 (96.7%)	1374 (96.6%)	1354 (95.2%)	
Other	61 (4.3%)	47 (3.31%)	48 (3.4%)	69 (4.8%)	
**Place of residence (*n*, %)**					*p* = 0.0476
Urban	927 (65.1%)	987 (69.5%)	945 (66.5%)	966 (67.9%)	
Rural	496 (34.9%)	433 (30.5%)	477 (33.5%)	457 (32.1%)	
**Smoking habits (*n*, %)**					*p* = 0.0261
Non-smokers	1028 (72.2%)	1041 (73.3%)	1067 (75.0%)	1094 (76.9%)	
Smokers	395 (27.8%)	379 (26.7%)	355 (25.0%)	329 (23.1%)	
**Parity (*n*, %)**					*p* = 0.2125
Nulliparity	832 (58.5%)	864 (60.8%)	763 (53.7%)	908 (63.8%)	
Multiparity	591 (41.5%)	556 (39.2%)	659 (46.3%)	515 (36.2%)	
**Pre-pregnancy BMI status (*n*,** **%)**					*p* < 0.0001
Normal weight	1021 (71.7%)	972 (68.4%)	1099 (77.3%)	1214 (85.3%)	
Overweight	294 (20.7%)	256 (25.1%)	257 (18.1%)	153 (10.8%)	
Obese	108 (7.6%)	92 (6.5%)	66 (4.6%)	56 (3.9%)	
**Gestational weight gain** **(mean ± SD; Kg)**	14.5 ± 6.5	14.1 ± 5.8	13.8 ± 6.3	13.4 ± 5.9	*p* < 0.0001
**Preterm birth (<37th week, *n*, %)**					*p* = 0.1943
No	928 (65.2%)	1035 (72.9%)	945 (66.5%)	1076 (75.6%)	
Yes	495 (34.8%)	385 (27.1%)	477 (33.5%)	347 (24.4%)	
**Gestational diabetes (*n*, %)**					*p* < 0.0001
No	1269 (89.2%)	1307 (92.0%)	1362 (95.8%)	1378 (96.8%)	
Yes	154 (10.8%)	113 (8.0%)	60 (4.2.%)	45 (3.2%)	
**Gestational hypertension (*n*, %)**					*p* = 0.2011
No	1362 (95.7%)	1346 (94.8%)	1397 (98.2%)	1350 (94.9%)	
Yes	61 (4.3%)	74 (5.2%)	25 (1.8%)	73 (5.1%)	
**Type of delivery (*n*, %)**					*p* < 0.0001
Vaginal	477 (33.5%)	540 (38.0%)	652 (45.9%)	833 (58.5%)	
Caesarean section	946 (66.5%)	880 (62.0%)	770 (54.1%)	590 (41.5%)	
**Exclusive breastfeeding (*n*, %)**					*p* < 0.0001
No	1204 (84.6%)	950 (66.9%)	468 (32.9%)	252 (17.7%)	
Yes	219 (15.4%)	470 (33.1%)	954 (67.1%)	1171 (82.2%)	

**Table 3:** Multivariate regression analysis for adherence to the MD.

Characteristics	Mediterranean Diet Adherence (Very Low + Low vs. Moderate + High)	
	OR * (95% CI **)	*p*-Value
**Age** (below/over mean value)	1.28 (1.94–1.52)	*p* = 0.0021
**Education level** (Primary and secondary education/university studies)	1.88 (1.61–2.12)	*p* = 0.0032
**Family economic status** (low or medium/high)	1.36 (0.95–1.69)	*p* = 0.0287
**Nationality** (Greek/other)	0.89 (0.28–1.41)	*p* = 0.2394
**Type of residence** (Urban/rural)	1.12 (0.61–1.78)	*p* = 0.2084
**Smoking habits** (no/yes)	1.12 (0.68–1.71)	*p* = 0.2298
**Parity** (nulliparity/multiparity)	1.07 (0.62–1.68)	*p* = 0.3986
**Pre-pregnancy BMI status** (overweight and obese/normal weight)	2.15 (1.92–2.38)	*p* = 0.0001
**Gestational weight gain** (over/below mean value)	1.78 (1.51–2.02)	*p* = 0.0007
**Preterm birth** (yes/no)	0.97 (0.35–1.67)	*p* = 0.5921
**Gestational diabetes** (yes/no)	2.32 (2.13–2.57)	*p* = 0.0004
**Gestational hypertension** (yes/no)	0.94 (0.23–1.71)	*p* = 0.6732
**Type of delivery** (caesarean section/vaginal)	1.89 (1.62–2.13)	*p* = 0.0003
**Exclusive breastfeeding** (no/yes)	2.45 (2.26–2.63)	*p* = 0.0001

* Odds Ratio: OR; ** CI: confidence interval.

## Data Availability

Data are available upon request from the corresponding author.
